# Short-term effects of argon cold atmospheric plasma on canine corneas *ex vivo*

**DOI:** 10.3389/fvets.2025.1518071

**Published:** 2025-02-11

**Authors:** Juliane Dick, Sandra Lockow, Wolfgang Baumgärtner, Holger Andreas Volk, Claudia Busse

**Affiliations:** ^1^Clinic for Small Animals, University of Veterinary Medicine, Foundation, Hannover, Germany; ^2^Department of Pathology, University of Veterinary Medicine, Foundation, Hannover, Germany

**Keywords:** cold atmospheric plasma, plasma pen, cornea, epithelization, dogs, air-liquid

## Abstract

**Purpose:**

To analyse the effects of argon cold atmospheric plasma (CAP) on canine corneas.

**Methods:**

Healthy canine eyes (*n* = 20) were subjected to a keratectomy (5 × 7 mm, 400 μm) and divided into two control (c1—not cultured; t0—cultured) and two treatment groups (t2, t5—treatment of 2 or 5 min, cultured); *n* = 5 eyes each. The kINPen^®^ VET (neoplas GmbH, Greifswald, Germany) was used for CAP treatment. Corneas (t0, t2, t5) were cultured at an air-liquid interface (72 h). Histopathological and immunohistochemical (Ki-67, Caspase-3, α-SMA) examinations were performed.

**Results:**

Corneal epithelization was complete and epithelial thickness was similar in all eyes. The number of perilimbal epithelial cell nuclei varied between groups with c1 = 22 ± 6, t0 = 13 ± 5, t2 = 15 ± 5 and t5 = 10 ± 4 nuclei/randomized fields and was lowest in t5, which was significantly different from t2 but not from t0. Ki-67 positive cells in the stroma varied between groups with c1 = 0.2 ± 0.45, t0 = 8 ± 12, t2 = 18 ± 12 and t5 = 10 ± 7 positive cells/section. More Ki-67 positive cells were found in t2 compared to t5. This was not significantly different from t0. Caspase-3 and α-SMA expression were similar in all treatment groups.

**Conclusion:**

Canine corneas treated with CAP showed similar corneal wound healing compared to untreated corneas *ex vivo*. A 5-min CAP application results in a lower perilimbal epithelial cell density and fewer Ki67 positive stromal cells compared to the 2-min treatment.

## Introduction

Infectious keratitis is one of the most common and potentially sight or even globe threatening ocular disease in human and veterinary ophthalmology (1, 2). In dogs, it is usually caused by bacterial infection and a number of different species have been identified as causative agents (3, 4). While the therapy of infectious keratitis in dogs is not standardized it always includes frequent topical antibiotics (5–7).

However, none of the available antibiotics covers all microorganisms possibly involved, making empirical therapy prior to culture and sensitivity results challenging (3, 6, 8). The increasing resistance of pathogenic microorganisms to antibiotics and the increasingly regulated use of antimicrobials in veterinary medicine in the European Union (Regulation (EU) 2019/6), make the choice of therapy increasingly difficult (6–9). There is a need for antimicrobial treatment alternatives for infectious keratitis to improve our treatment success but also to conquer the increasing challenges around the use of antibiotics in human as well as veterinary medicine (2, 3, 8, 10, 11).

Cold atmospheric plasma (CAP), is an ionized gas, composed of electrons, ions, ultraviolet radiation, electromagnetic fields, reactive oxygen (ROS) and nitrogen species (RNS) (12). It is described as “low temperature” or “cold” plasma, because of its temperature of only about up to 40° C, which is close to body temperature and allows direct application on living tissue without anesthesia (13). Different physical methods are available to generate CAP (12, 13). The plasma device of the kINPen^®^ VET (neoplas GmbH, Greifswald, Germany) generates CAP through a needle electrode that is surrounded by a capillary and a ring electrode (13). Argon flows through the capillary and acts as a carrier gas for the plasma (13). On the tip of the plasma pen the plasma becomes visible in form of a bluish to purple “plasma jet,” allowing an exact application (10, 12).

CAP is increasingly used in medicine for its anti-inflammatory and wound healing effects (14–18) and is gaining increasing interest because of its antimicrobial properties, which include efficacy against multidrug-resistant pathogens (14, 19). ROS, RNS and ultraviolet radiation are considered to be the main players in plasma-induced biological outcomes (13, 20). The bactericidal effects are caused by membrane oxidation as well as cell wall and DNA disintegration in prokaryotes (13). Reitberger et al. (10) found that *ex vivo* corneas artificially infected with *Staphylococcus aureus* demonstrate no bacterial growth after a 5-min CAP treatment. CAP treatment also reduced bacterial load and ulcer formation of leporine corneas *in vivo* which have been artificially infected with *Pseudomonas aeruginosa* (21). Marx et al. (22) showed that CAP application *in vitro* inhibits bacterial growth of the pathogens most commonly involved in canine infectious keratitis (*Pseudomonas aeruginosa, Staphylococcus pseudintermedius*, and *Streptococcus canis*). In future, CAP may be a new addition in the treatment of corneal infections (10).

Its potent antimicrobial properties, easy and painless application under local anesthesia as well as potential positive impact on wound healing make CAP an interesting tool for ophthalmic patients, warranting further investigations on its effects on ocular tissue (10, 12, 18).

The aim of this study was to investigate the short-term effects of CAP treatment on canine corneas in an *ex vivo* organ culture model.

## Materials and methods

### Collection of canine corneas

The donor corneas were harvested from dogs, which were euthanized for reasons unrelated to this study. Signalment and clinical history were recorded (Table 1). Both eyes were examined using slitlamp biomicroscopy (SL-17, Hand-Spaltlampe LED, KOWA Europe GmbH, Tokyo, Japan). The eyes were enucleated within 8 h after the time of death. Subsequently, the eyes were stored in a cooled, sterile phosphate-buffered saline (PBS) or in a 0.9% isotonic saline solution.

**Table 1:** Signalment and clinical history of the dogs that donated corneas.

**Sample number**	**Patient number**	**Age (years)**	**Sex**	**Breed**	**Reason for euthanasia**
E15	T/0439759	0.5	Male	Lagotto romagnolo	Cerebellar abiotrophy
E16	T/0439759	0.5	Male	Lagotto romagnolo	Cerebellar abiotrophy
E17	T/0440297	7	Female	Mixed breed	Neurological disorder
E18	T/0440297	7	Female	Mixed breed	Neurological disorder
E19	T/0423509	11	Male	Labrador retriever	Anal pouch carcinoma
E20	T/0423509	11	Male	Labrador retriever	Anal pouch carcinoma
E21	T/0435113	11	Female, neutered	Labrador retriever	Hemangiosarcoma
E22	T/0435113	11	Female, neutered	Labrador retriever	Hemangiosarcoma
E23	T/0443819	6	Male	Dachshund	L4-S1 myelopathy, paraplegia
E24	T/0443819	6	Male	Dachshund	L4-S1 myelopathy, paraplegia
E25	T/0447110	6	Female, neutered	Labrador retriever	L4-S1 myelopathy, paraparesis
E26	T/0447110	6	Female, neutered	Labrador retriever	L4-S1 myelopathy, paraparesis
E27	T/0452272	13.75	Female, neutered	Giant schnauzer	Central vestibular syndrom, euthyroid sick syndrome
E28	T/0452272	13.75	Female, neutered	Giant schnauzer	Central vestibular syndrom, euthyroid sick syndrome
E29	T/0452693	7.75	Male	Jack Russell terrier	Discopathy
E30	T/0452693	7.75	Male	Jack Russell terrier	Discopathy
E31	T/0452994	8.25	Female	Bavarian mountain hound	Myelopathy, hemilaminectomy
E32	T/0452994	8.25	Female	Bavarian mountain hound	Myelopathy, hemilaminectomy
E33	n.a.	13	Female	Mixed-breed terrier	Dementia
E34	n.a.	13	Female	Mixed-breed terrier	Dementia
E35	T/0456834	13	Male	Maltese	Paralysis, paresis
E36	T/0456834	13	Male	Maltese	Paralysis, paresis

n.a., not applicable.

**Figure 1:**
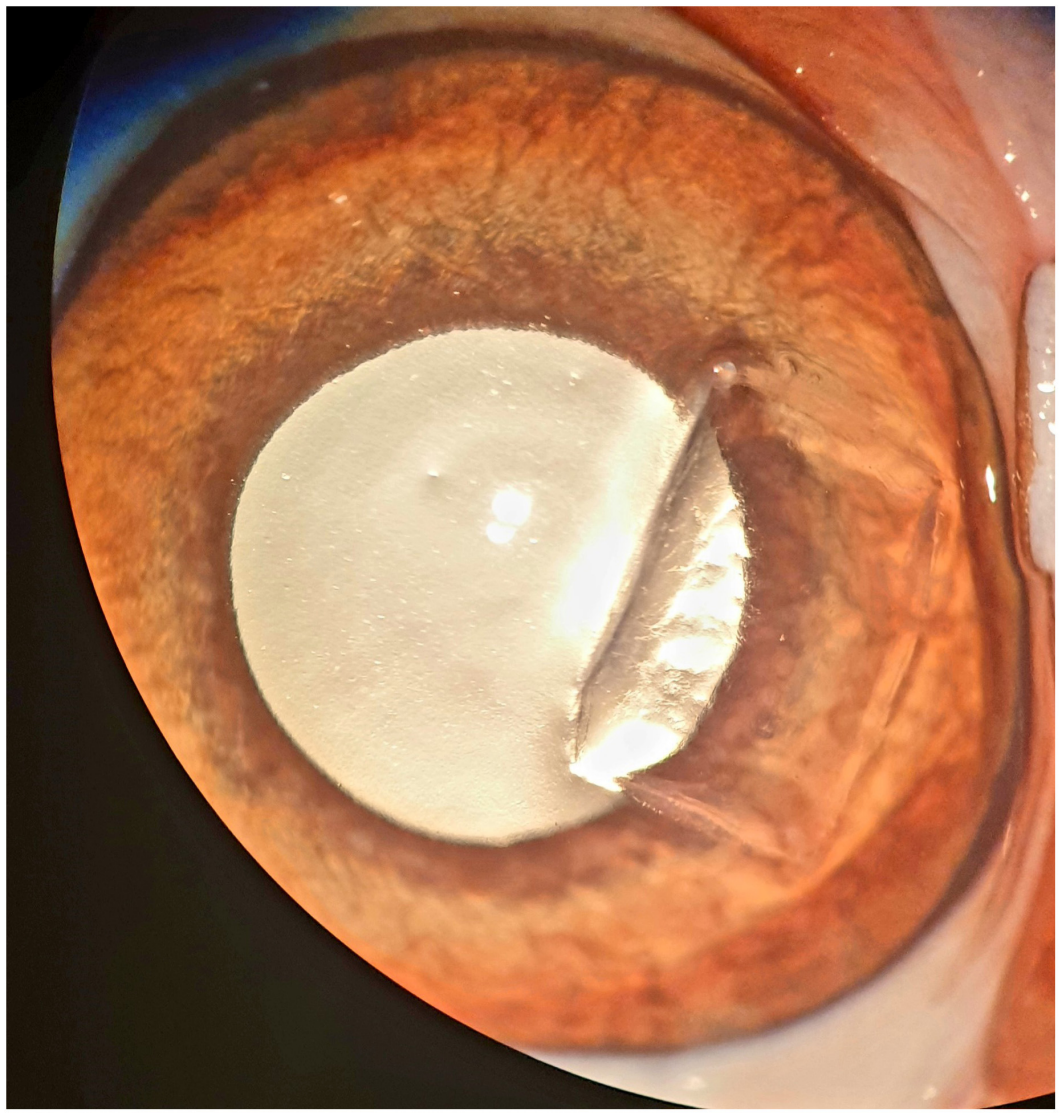
Canine cornea following a superficial keratectomy, 5 × 7 mm in diameter, control group c1 (E15; see Table 4).

A superficial lamellar keratectomy was performed on each cornea under an operating microscope (Carl Zeiss OPMI Visu 210 S8, Carl Zeiss, Oberkochen, Germany). A stromal defect of 5 mm width and 7 mm length (Figure 1) was outlined using a set depth knife (Restricted Depth Knife Straight, 300 Micron, Surgical Specialties Corporation, Sharepoint, Westwood in Los Angeles, USA) which defined the stromal depth of 300 μm. The outlined cornea was then removed using a crescent knife (Crescent Knife, LaserEdge Plus, Bausch& Lomb incorporated, Rochester in New York, USA). The corneas were moistened repeatedly during surgery with the transport medium (either PBS or 0.9% isotonic saline solution).

**Figure 2:**
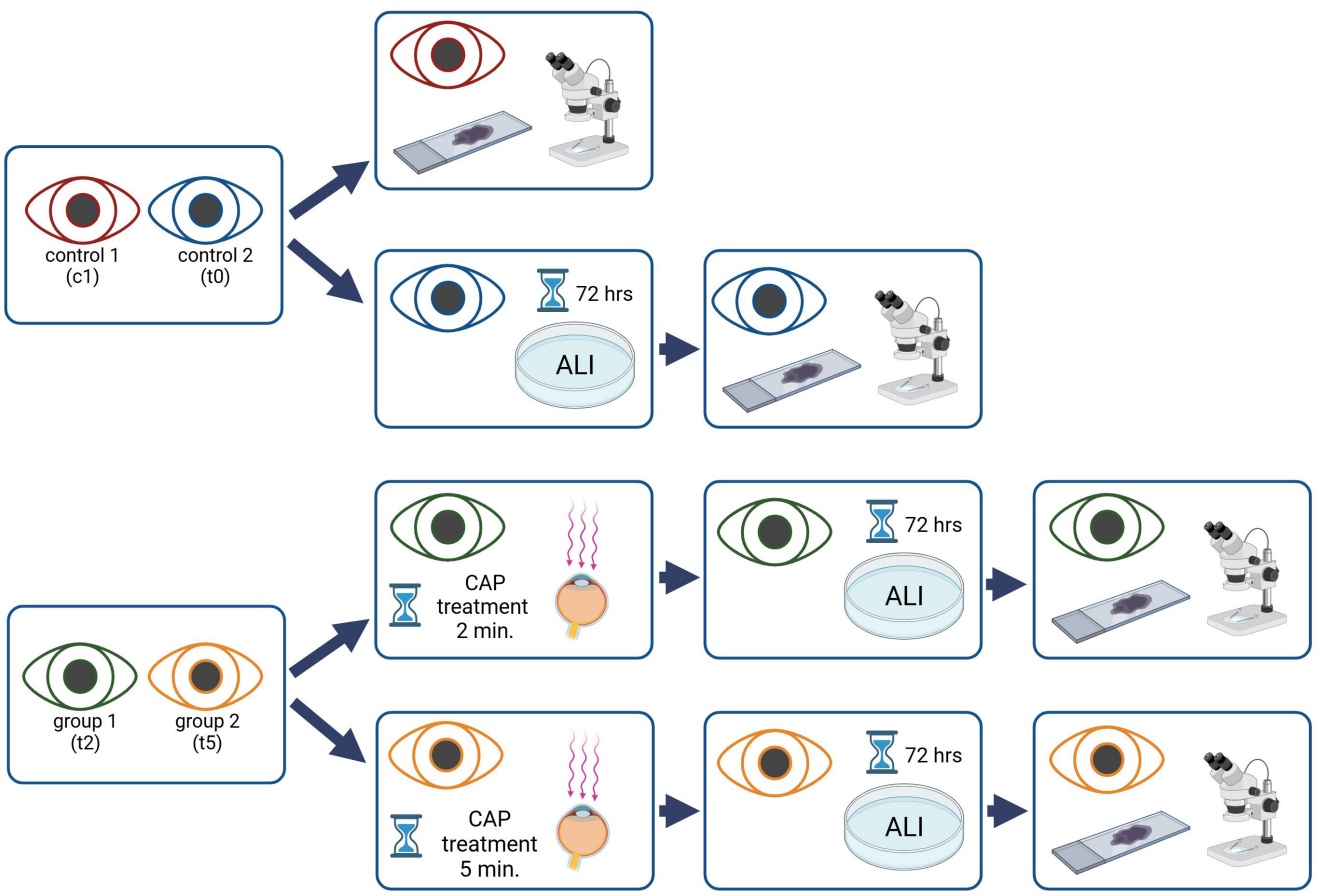
Flow chart, overview of the study procedure, the eyes of one dog were used either as control or as CAP treated samples. Created with Biorender.com.

Eyes were divided into two control (c1 and t0) and two treatment groups (t2 and t5). The eyes in the control group were not treated with CAP. Eyes in group c1 were immediately processed, while eyes in group t0 were cultured for 72 hours as described below before also being processed. The eyes of the treatment groups were treated with CAP for 2 min (group t2), or 5 min (group t5; Figure 2). All eyes treated with CAP (t2 and t5) were cultured for 72 h.

### Air-liquid-interface-cultivation

**Figure 3:**
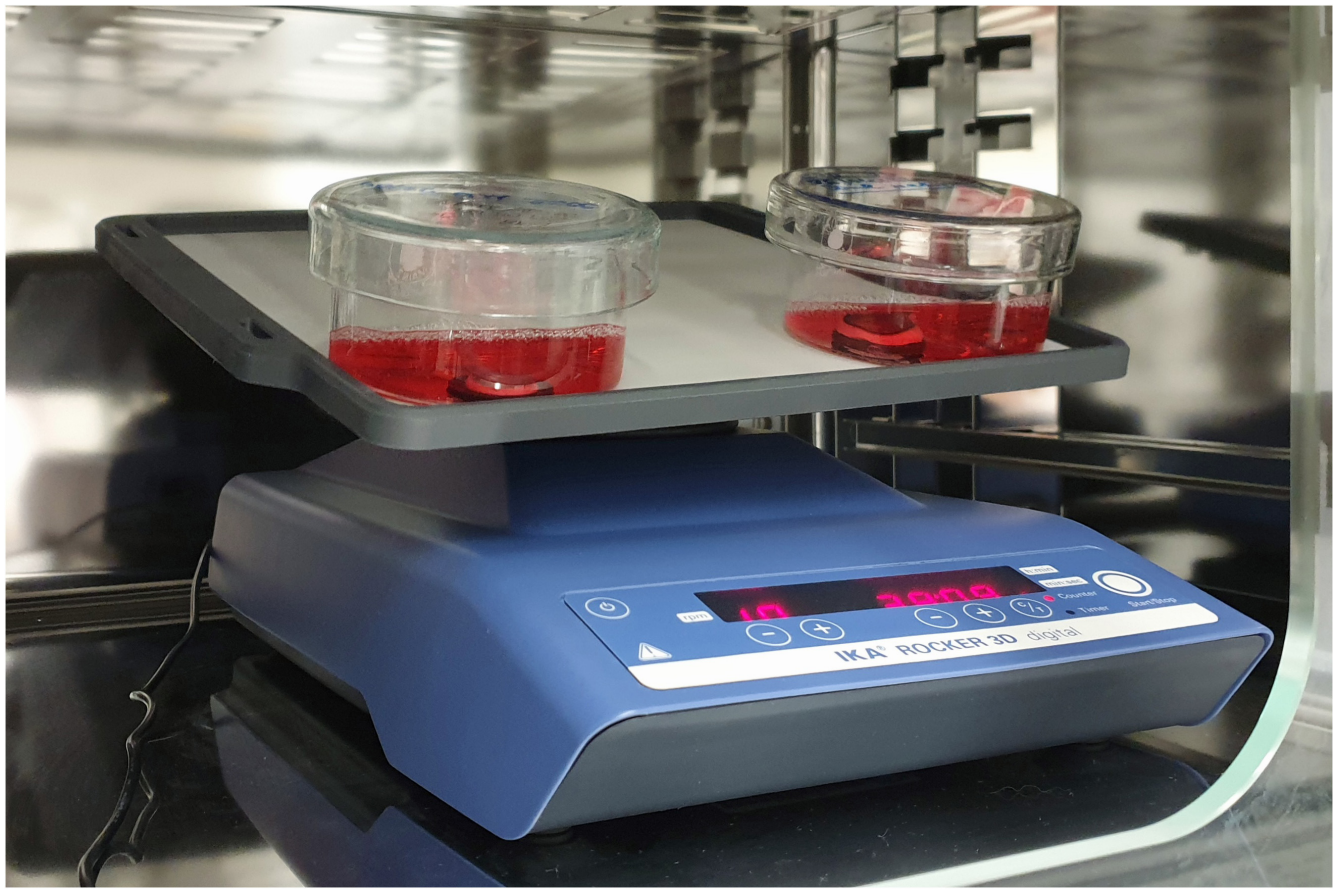
ALI culture of two corneas: the agarose- filled corneoscleral buttons are placed on sealing rings, which were fixed in the petri dishes. The rocking platform is placed in an incubator with defined milieu: 37°C and 5% CO_2_, the natural environment of corneas is imitated.

The ALI culture of the corneas of the group t0, t2 and t5 was performed as described previously (23). Few modifications were applied: First; the heat-resistant sealing rings (3/4 Zoll, Manufaktur Martinshof, Niesky, Germany) were fixed using surgical tissue adhesive (Histoacryl^®^, B. Braun Surgial, S.A., Rubí, Spain). Second; each cornea was washed with cooled PBS and disinfected twice with polyhexanide solution (Lavanid^®^ 2 wound irrigation solution, SERAG WIESSNER, Naila, Germany), for 2 min, before being placed in culture (24). The volume of the medium varied from 20 to 30 ml to correlate with the dimensions of the petri dish and the cornea. Third, the medium was changed every 2 days. Corneas were cultured for 72 h (Figure 3). The ALI culture was established prior to the study with separate sets of paired eyes of five dogs of which one eye each was processed immediately and the other was cultured for 72 h in three cases and for seven days in one case and then processed (results not shown). Results were similar with those of Harman et al. confirming that our ALI culture was functional and no pathological changes were observed in the corneal epithelium and stroma.

### Cold atmospheric plasma

**Figure 4:**
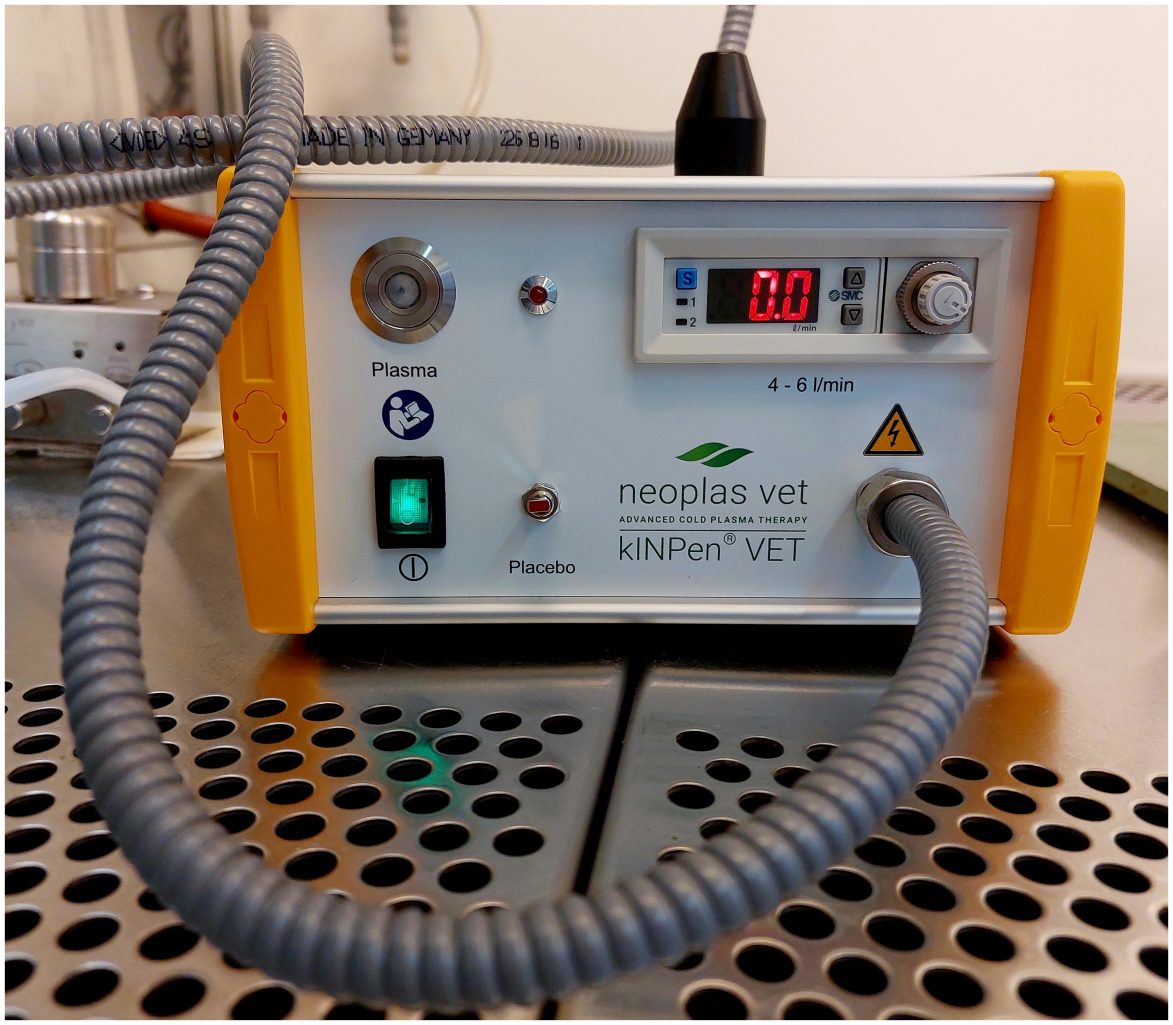
Plasma device kINPen^®^ VET.

**Figure 5:**
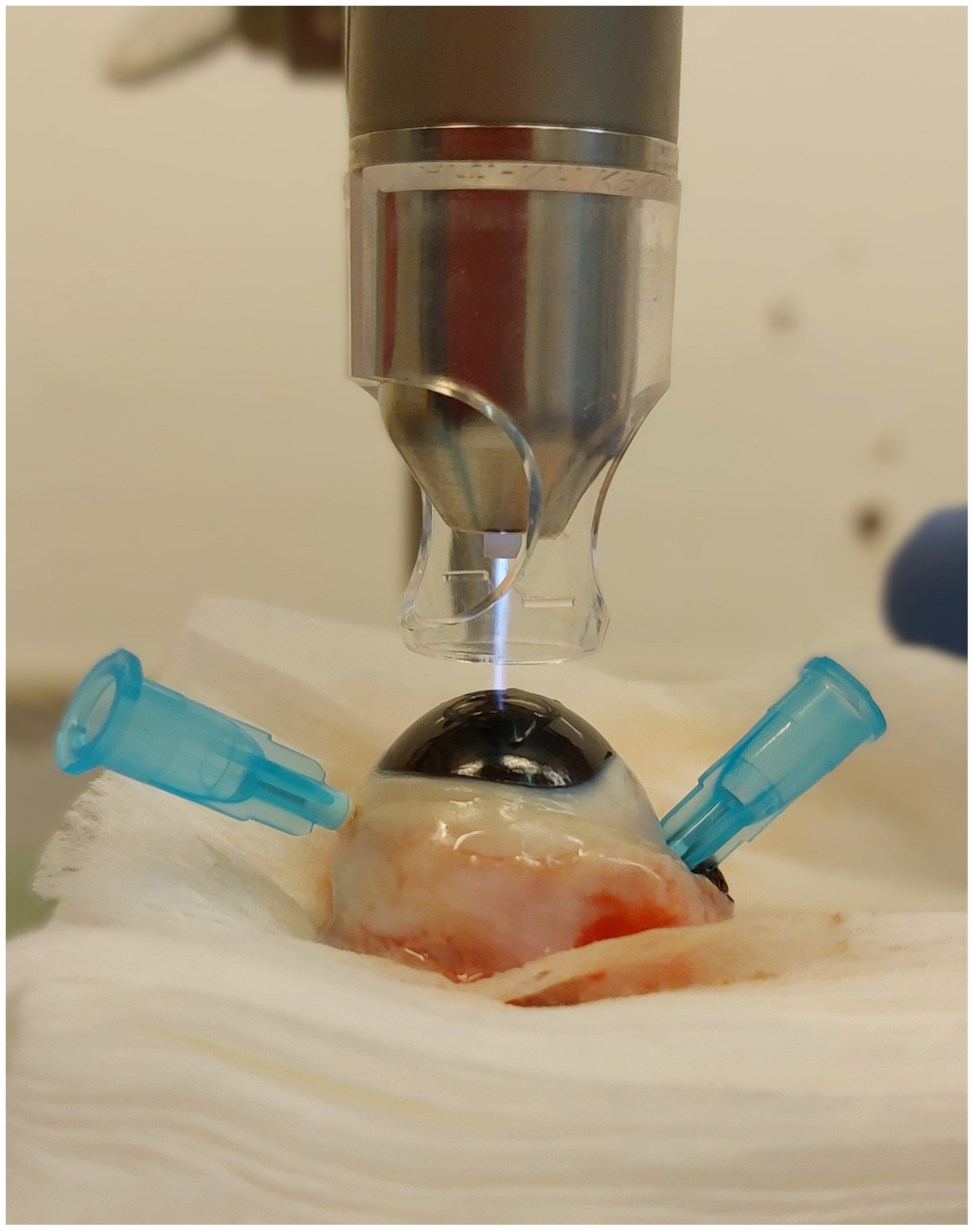
Plasma treatment of a cornea in conducting mode, with spacer, the plasma jet touches the corneal surface.

For this study the argon plasma generator kINPen^®^ VET (neoplas GmbH, Greifswald, Germany, Figure 4) was used. A transparent spacer (neoplas GmbH, Greifswald, Germany) was placed at the tip of the plasma pen, to focus the plasma jet and to maintain the distance between the pen and the tissue (Figure 5).

**Figure 6:**
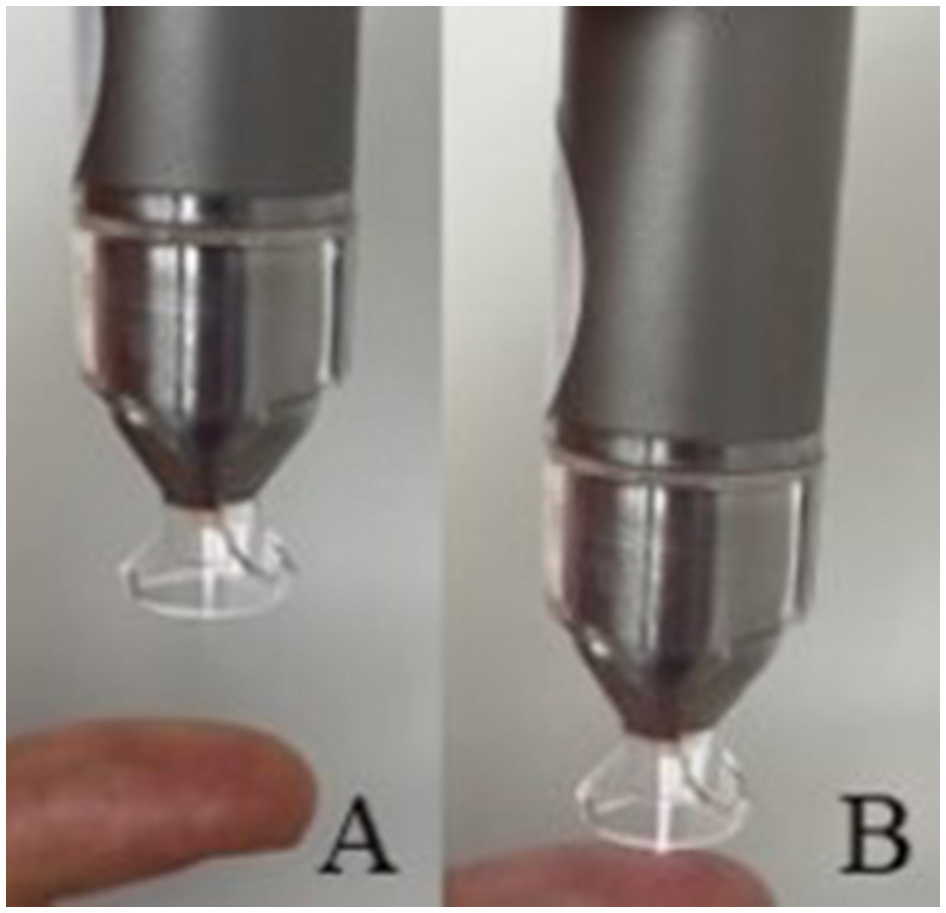
**(A)** Plasma jet in non-conducting and **(B)** in conducting mode, note the visible reaction between the finger and the plasma jet.

CAP treatment of the corneas (group t2 and t5) was performed immediately following the keratectomy. The eyes were fixed with 23-gauge cannulas. One eye each of the same dog was randomly assigned to treatment group t2 and group t5. The plasma pen was fixed in a scaffold so that the distance between the corneal surface and the spacer was between 2 and 3 mm. This distance allowed a continuous plasma treatment in “conducting mode” (25). This means that the visible tip of the plasma jet reached the corneal surface (Figure 5), leading to a visible reaction with the tissue surface in form of a more intensive plasma jet (Figure 6). The plasma flow was set at 4.1 L/min, which was the lowest flow rate, that allowed plasma generation in the non-modified kINPen^®^ VET. Corneas were moistened with PBS immediately before and after plasma treatment. During treatment the Petri dish was moved manually in a meandering pattern to allow for a uniform plasma distribution. CAP treatment time intervals were either two (group t2) or 5 min (group t5). Treated eyes were stored in PBS until further preparation for ALI cultivation. After 72 h of culture, the corneoscleral buttons were removed from the culture medium, bisected with a microtome blade in the center of the keratectomy and placed in 10% paraformaldehyde for 24 h.

### Histology

**Figure 7:**
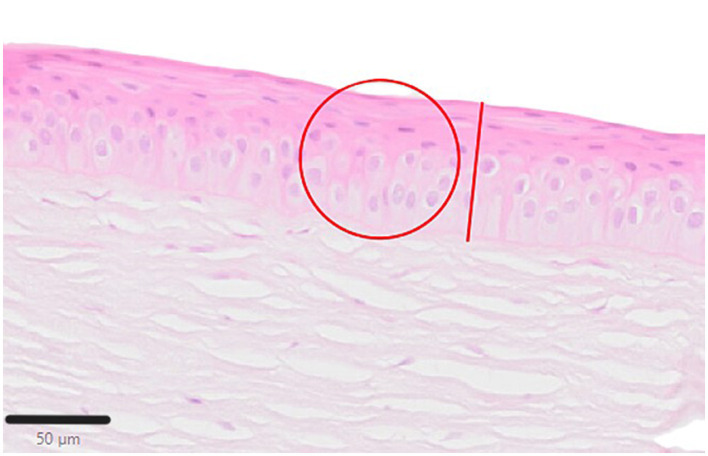
Schematic illustration for sample evaluation. The red line demonstrates the measurement of the epithelial layer thickness. The cell nuclei were counted within the red defined circle, H&E; Scale bar, 50 μm.

The paraffin-embedded corneas were sectioned in 2 μm slices and fixed on glass slides before being stained routinely with hematoxylin and eosin (H&E) and periodic acid-Schiff reaction (PAS) (26). The stained samples were scanned (Olympus VS200 slide scanner, Olympus Deutschland GmbH, Hamburg, Germany) and analyzed using QuPath-0.4.2 (QuPath open software for bioimage analysis, licensed under the GNU General Public License) (27). Samples were evaluated as follows: Epithelial thickness was measured at five locations (twice near the limbus, at either end and in the center of the keratectomy site). In these areas, the cell nuclei were also counted in a defined circular area with a diameter of 80 μm (acreage of ~50,276.5μm^2^; Figure 7).

### Immunohistochemistry (IHC)

An immunohistochemical analysis was conducted on formalin-fixed, paraffin-embedded samples. The investigated antibodies were caspase-3 (Cas-3), Ki-67 and alpha-smooth muscle actin (α -SMA) (28). Other canine tissues (Cas-3 → lymph node; Ki-67 → small intestine; α -SMA → musculature) were used as positive controls. The evaluation was performed under a light microscope (Olympus BX53 system microscope, Olympus lifescience/Evident, Tokyo, Japan) with 200 × and 400 × magnification. Photographs of relevant sections were taken with a digital microscope camera (Olympus DP72 camera, Olympus lifescience/Evident, Tokyo, Japan). Cas-3 and Ki-67 positive stained nuclei and cytoplasm were counted totally in both, epithelium and stroma. The counting area was defined from limbus to limbus, including the part of the keratectomy. The α-SMA positive stained cells were counted in five visual fields per sample in epithelium and stroma. The stroma was divided visually into thirds from anterior to posterior.

### Statistical analysis

Statistical analyses were performed with SAS Enterprise Guide 7.1 (SAS Institute Inc., Cary in North Carolina, USA) using the linear mixed (continuous variables) and the mixed Poisson model (counting data). *p*-values of equal to or <0.05 was considered statistically significant.

## Results

The ALI culture was uneventful with no evidence of secondary bacterial infection in any sample. The corneas became slightly edematous after 1 day of cultivation, and this condition remained unchanged until the end of the cultivation period. During treatment we noted that the plasma jet had a drying effect on the cornea.

### Histological evaluation

**Figure 8:**
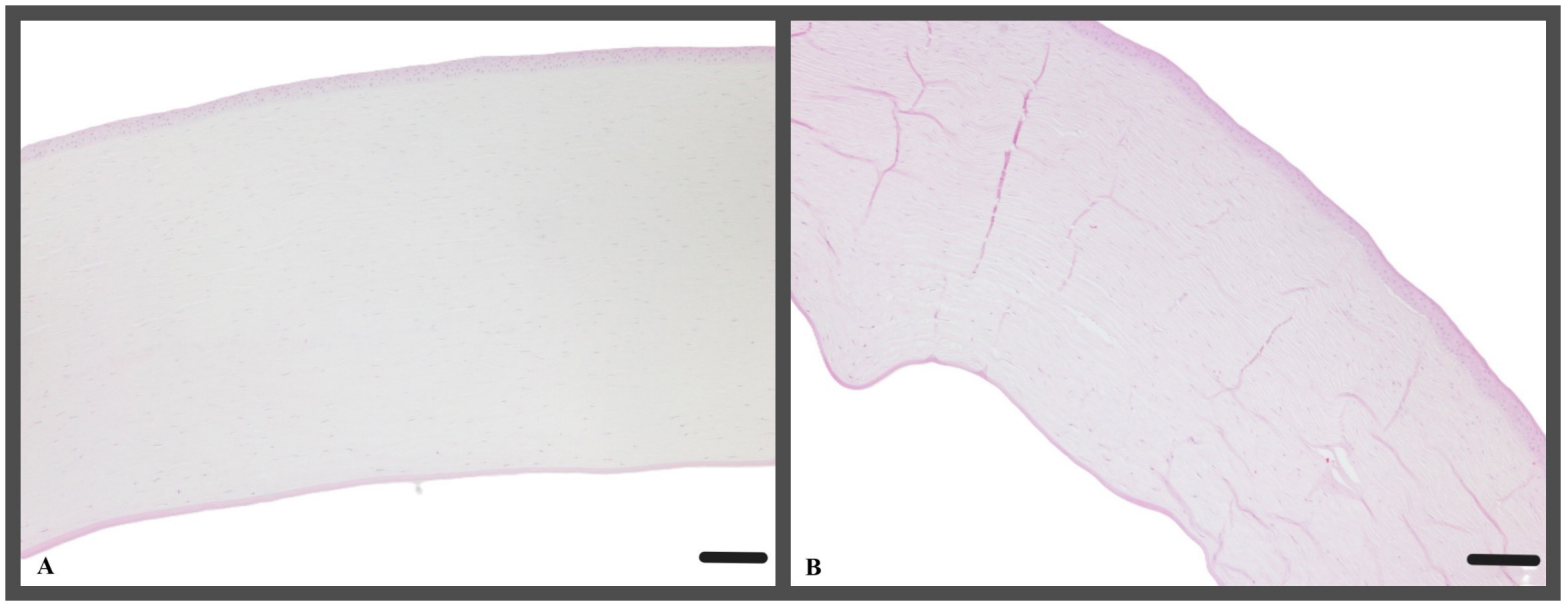
**(A)** Representative example of a cornea, that was treated with CAP for 2 min (t2, canine cornea, followed by 72 h ALI cultivation); H&E; Scale bar, 200 μm and **(B)** representative example of a cornea, treated with CAP for 5 min (t5, canine cornea, followed by 72 h ALI cultivation), stromal folds are fixation artifacts; perilimbal area, keratectomy is not shown, H&E; Scale bar, 200 μm.

**Figure 9:**
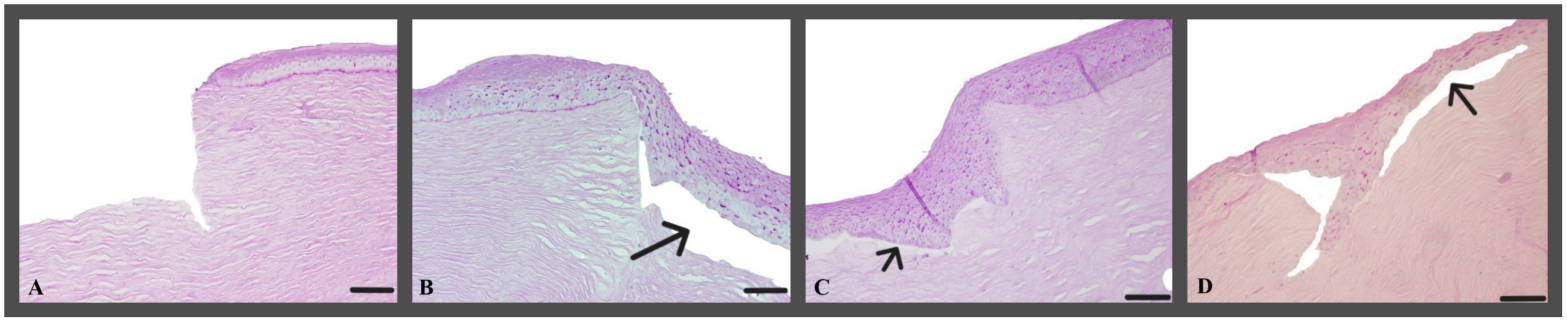
**(A–D) (A)** Canine cornea after keratectomy without cultivation (c1, canine cornea, no CAP treatment); **(B–D)** continuous epithelial layer, keratectomised area with hyperplastic epithelium, gap between epithelium and stroma (arrows) represent an artifact caused by the processing of the tissue. **(A)** c1, canine cornea, no CAP treatment; **(A)** t0, canine cornea, followed by 72 h ALI cultivation, no CAP treatment; **(C)** t2, canine cornea, followed by 72 h ALI cultivation, 2-min CAP treatment; **(D)** t5, canine cornea, followed by 72 h ALI cultivation, 5-min CAP treatment; notice lack of microscopic changes regardless of the pre-treatment; PAS; Scale bar, 100 μm.

Histological analyses of the corneas in H&E and PAS-stained sections revealed a consistent cell morphology across the corneal surface (Figure 8). Unlike the control group c1, where the corneas were not cultivated, all other corneas (t0, t2, t5) exhibited a continuous epithelium, including the keratectomised area, after 72 h of ALI culture. The epithelial layer in the keratectomised area was significantly thicker, when compared to the perilimbal epithelium in all cultured corneas (Figure 9, Table 2). When comparing two corneas of the same dog, one treated with CAP for 2 min and 5 min, respectively, revealed no consistent changes of cellular differences.

**Table 2:** Corneal epithelial thickness of the control (c1, t0) and CAP treatment groups (t2, t5) was measured in three areas as mean value ± standard deviation (μm).

	**Epithelial thickness lim (μm)**	**Epithelial thickness ek (μm)**	**Epithelial thickness ck (μm)**
Control 1 (c1)	68.24 ± 9.49	n.a.	n.a.
Control 2 (t0)	58.15 ± 17.33	141.88 ± 54.98	195.71 ± 37.84
Treatment 1 (t2)	59.04 ± 16.03	166.35 ± 108.67	95.64 ± 33.81
Treatment 2 (t5)	44.45 ± 25.09	176.45 ± 133.99	100.29 ± 99.83

C1 was analyzed without ALI cultivation. T0, t2 and t5 were cultivated for 72 hurs. The duration of CAP application was set to 2 min (t2) and 5 min (t5).

Lim, perilimbal; ek, edge of keratectomy, ck = center of keratectomy; n.a. = not applicable post keratectomy.

Epithelial thickness was not significantly different across all groups (Table 2).

Quantitative analysis of corneal epithelial cell nuclei did not show a significant difference between cultured corneas. However, the density of nuclei in the perilimbal region was lowest after the 5-min plasma treatment, averaging 10 ± 4 nuclei per selected area, particularly when compared to t2 with ~15 ± 5 nuclei/selected area (*p* = 0.0422; Table 3). The nucleus density was highest in c1 with 22 ± 6 nuclei/selected area, compared to t0 (*p* = 0.0109) and t5 (*p* = 0.0038). In t0 and t2, the cell density exhibited similar behavior, with 13 ± 5 nuclei/selected area (t0) and 15 ± 5 nuclei/selected area (t2). No significant differences of the density of nuclei were detected between t0 and t2 and between t0 and t5.

**Table 3:** Number of corneal epithelial cell nuclei/selected area (= area with an acreage of 50,276.5 μm^2^ here defined as epithelial cell density) of the control (c1, t0) and treatment groups (t2, t5) as mean value ± standard deviation.

	**Epithelial cell density lim (nuclei/selected area)**	**Epithelial cell density ek (nuclei/selected area)**	**Epithelial cell density ck (nuclei/selected area)**
Control 1 (c1)	22 ± 6ab	n.a.	n.a.
Control 2 (t0)	13 ± 5a	13 ± 3	15 ± 7
Treatment 1 (t2)	15 ± 5c	12 ± 2	17 ± 2
Treatment 2 (t5)	10 ± 4bc	13 ± 4	9 ± 7

T0, t2 and t5 were cultivated for 72 h. The duration of CAP application was set to 2 min (t2) and 5 min (t5).

Lim, perilimbal; ek, edge of keratectomy; ck, center of keratectomy; n.a., not applicable post keratectomy. a = p = 0.0109, b = p = 0.0038, c = p = 0.0422.

### Immunohistochemistry

#### Ki 67

**Figure 10:**
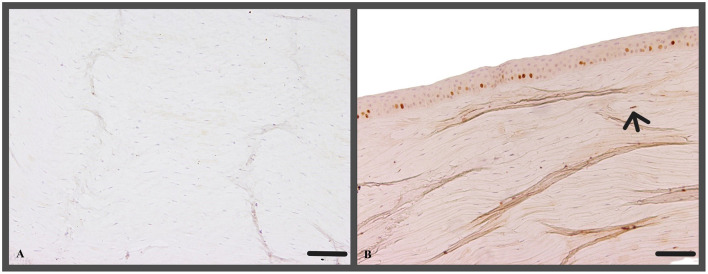
**(A)** No Ki-67 positive cells within the stroma (c1, canine cornea, no CAP treatment, no ALI culture); Ki-67 IHC; Scale bar, 100 μm; **(B)** Ki-67 positive nuclei of stromal cell (arrow, t5, canine cornea, 5 min CAP application, ALI culture for 72 h); Ki-67 IHC; Scale bar, 100 μm.

In the epithelium the evaluation of Ki-67 positive cells did not reveal significant differences among the two treatment groups nor when compared to the control group, t0 (Figure 10A). However, in the stroma a significantly higher number of Ki-67 positive cells were found in t2 (18 ± 12 Ki-67 positive cells/section) corneas compared to group t5 (10 ± 7 Ki-67 positive cells/section; *p* = 0.0111; Figure 10B).

#### Alpha-SMA

**Figure 11:**
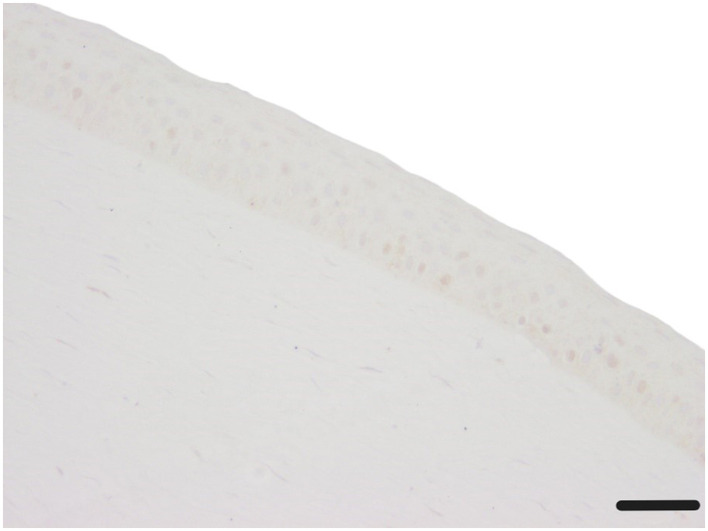
Corneal epithelium and stroma without any α-SMA stained cytoplasm (c1, canine cornea, no CAP treatment, followed by 72 h ALI cultivation); α-SMA IHC; Scale bar, 50 μm.

Alpha-SMA showed only sporadic positive staining in epithelium and stroma (0.2–0.8 positive α-SMA stained cytoplasm in epithelium and stroma), with no significant differences observed between the treatment groups (Table 4, Figure 11).

**Table 4:** Immunohistochemistry: Ki-67, caspase-3 and α-SMA of the control (c1, t0) and CAP treatment groups (t2, t5) as mean ± standard deviation.

	**Positive Ki-67-stained nuclei in epithelium from limbus to limbus**	**Positive Ki-67-stained nuclei in the stroma from limbus to limbus**	**Positive caspase-3-stained cytoplasm in epithelium from limbus to limbus**	**Positive caspase-3-stained cytoplasm in the stroma from limbus to limbus**	**Positive α-SMA-stained cytoplasm in epithelium from limbus to limbus**	**Positive α-stained cytoplasm in Stroma from limbus to limbus**
Control 1 (c1)	111 ± 31.63	0.2 ± 0.45abc	0	0	0	0.2 ± 0.45
Control 2 (t0)	92.80 ± 39.28	8.2 ± 12.32a	0.4 ± 0.55	0	0	0.4 ± 0.89
Treatment 1 (t2)	105.4 ± 29.7	17.6 ± 12.18bd	2.0 ± 1.22	0.2 ± 0.45	0	1.2 ± 2.23
Treatment 2 (t5)	114.2 ± 61.45	9.8 ± 6.94cd	2.4 ± 2.3	1.4 ± 1.67	0.2 ± 0.45	0.8 ± 1.79

In group c1, the corneas were not cultured and no CAP treatment was performed. The corneas of group t0 were not treated with CAP. The corneas of t0, t2 and t5 were cultured for 72 h using the ALI method. CAP application lasted 2 min for t2 corneas and 5 min for t5 corneas. The positively stained epithelial and stromal cells were counted in the entire tissue section.

a = p =0.0063, b = p = 0.0025, c = p = 0.0051, d = p = 0.0111.

#### Caspase 3

**Figure 12:**
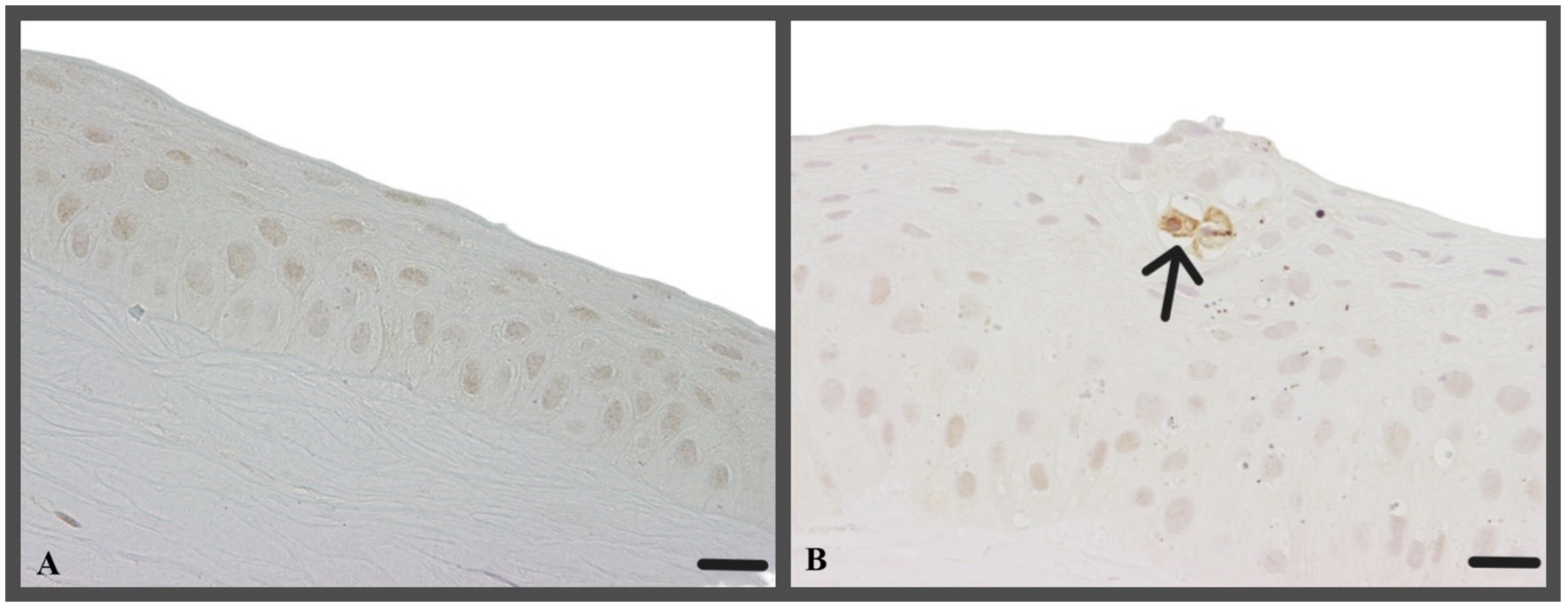
**(A)** Corneal epithelium and stroma without any Caspase-3-stained cell (t0, canine cornea, ALI cultivation for 72 h after keratectomy, no CAP treatment); Caspase-3 IHC; Scale bar, 20 μm; **(B)** Caspase-3-stained epithelial cell (arrow, t2, canine cornea, CAP treatment about 2 min, ALI culture for 72 h after keratectomy and CAP application); Caspase-3 IHC; Scale bar, 20 μm.

Caspase-3 positive cells were rarely detected in corneal epithelium (Table 4, Figure 12B). Corneas from t0, t2, and t5 exhibited slightly higher Caspase-3 staining in the epithelium compared to c1, but the observed differences were not statistically significant (*p* = 0.1220) (Figure 12A).

## Discussion

To our knowledge this is the first study looking at the effects of cold atmospheric plasma (CAP) on the canine cornea in an *ex vivo* corneal healing model. CAP treatment for two or 5 min had no detectable adverse effects on *ex vivo* epithelial wound healing.

Cultivating cells or organs like the cornea using air-liquid interface (ALI) culture is an effective method that mimics the natural environment of a mammalian cornea (29, 30). This technique involves alternating between liquid and air phases to replicate the blinking of eyelids, tear film distribution, and exposure to ambient air (29). ALI culture allows for studying the cornea as a three-dimensional organ and understanding the interactions between its different cell layers (epithelium, stroma and endothelium) (29, 31–33). Additionally, ALI culture enables the preservation of limbal stem cells and observation of cell migration during processes like wound healing (34). Foreman et al. (34) showed that bovine corneas can be completely re-epithelialized within 48 h of cultivation using this method. Berkowski et al. (32) described a full re-epithelization within 36–54 h in canine corneas after a stromal wound was created with stromal excimer laser phototherapeutic keratectomy. ALI culture serves as a valuable bridge between *in vitro* and *in vivo* experiments, eliminating the need for *in vivo* experiments and reducing animal testing within the framework of the 3 R concept (35).

In this research, corneas were cultured for 72 h. All samples displayed complete re-epithelialization of the keratectomised area in the histological assessment. While the epithelium exhibited hypertrophy in this region, the stroma did not show any abnormalities. The epithelial cell density of the cultured corneas t0 was notably lower near the limbus compared to the control corneas c1. The epithelial hypertrophy and the reduced perilimbal cell density after ALI cultivation are most indicative of the physiological processes in corneal wound healing where the epithelial cells migrate from the limbus to the ulceration to close the defect (5). Again, we used this method of cultivation to investigate the effects of CAP on living corneal tissue. CAP represents a new therapeutic option to promote the healing of corneal tissue. In human ophthalmology the disinfection potential of CAP is described without damaging corneal cells (10, 14). Reitberger et al. (10) described a safe CAP application on epithelial cells for 2–5 min. There was no change in apoptosis or oxidative stress markers, even after 5 min CAP treatment (10). Exposure for 10 min resulted in a reduction in viability with histologically visible damage to the cells (10). Brun et al. (14) described that a 2-min CAP treatment with helium as carrier gas inactivates ocular pathogens without causing significant tissue and DNA damage. After 5-min CAP treatment, the viability (MTT test) of stomal cells and fibroblasts decreased significantly (14).

The duration of CAP treatment in its current setting is limited by the drying effect causing visible desiccation damage to the corneal surface.

An evidence-based recommendation for a clinical treatment regime requires the consideration of several factors, such as the survival rate of the pathogens, the practical applicability of the treatment without general anesthesia and the safe application without damaging healthy cells. Reitberger et al. (10) described a case series of four patients with persistent infectious keratitis. CAP therapy was used as an adjuvant and reduced the inflammation of the ocular surface (10). The scar formation of the corneas and the visual capacity of the patients improved (10).

Here, all corneas treated with CAP for 2- (t2) or 5- (t5) min showed a similar corneal epithelial wound healing compared to untreated corneas. A noteworthy difference between groups was the lower epithelial cell density in the perilimbal region, after 5 min of CAP treatment compared to the other test groups. During wound healing, perilimbal epithelial cells migrate toward the corneal ulceration (5). The perilimbal cell density in group t5 was halved compared to group c1. This decreased cell density in the perilimbal area may be linked to an enhanced wound healing because the epithelial cells have already completed their migration to the ulceration. However, another interpretation could be that the limbal stem cells were inhibited resulting in a lower cell proliferation. As a consequence, the epithelial cells may not be reproduced in comparable numbers as in untreated corneas. A comprehensive study of the limbal stem cells will be necessary in the future to assess the impact of CAP on them.

To investigate cellular changes after ALI cultivation and CAP treatment, we chose several immunohistochemical marker.

Ki-67 is a protein that is commonly used as a biomarker in canine tissue and eyes to assess cell proliferation and tissue health (36, 37). It is expressed during the active phases of the cell cycle (36). In canine eyes it is an established biomarker to assess the corneal epithelial cell proliferation during cultivation (37). We could demonstrate normal corneal wound healing as illustrated by cell migration (5). The number of proliferating epithelial cells remained unchanged compared to the control group t0. However, there was a higher number of cell proliferations in the stroma of the cultivated corneas than in the c1, which indicates the cell proliferation during wound healing. Epithelial cell proliferation, after CAP treatment was similar in all groups. In the stroma, the 2-min CAP treatment compared to the 5-min treatment, resulting in a higher number of Ki-67 positive stromal cells, indicatin**g** an increased cell proliferation. We therefore presume that CAP can promote cell proliferation when used for a period of 2 min. Other studies concluded that CAP promotes the proliferation of fibroblasts, osteoblasts and haematopoietic stem cells in humans (38–40).

Caspase-3s role in apoptosis and its increased activity in response to cellular damage make it a valuable tool for early detection and monitoring of disease progression (41, 42). It was also detected in corneal epithelium of rats after UV-B irradiation and in conjunctival cells of rabbits with dry-eye-syndrome (43, 44). Immunohistochemistry evaluation of epithelium and stroma revealed that there was no increase in apoptosis (caspase-3) as a result of the cultivation. There was also no significant caspase activation in any of our CAP treatment groups. Weiss et al. (45) also found, that there was no dose-dependent increase of caspase 3 and 7 after CAP treatment of human fibroblasts.

A-SMA has emerged as a valuable biomarker in canine tissue for assessing fibrosis and myofibroblast activation (46). Its expression is indicative of tissue remodeling and repair processes, including scar formation (47). In this context, it is also used to assess corneal wound healing (32, 46). Here, the evaluation of epithelium and stroma showed that there was no cellular damage as a result of the cultivation. The number of fibrotic (α-SMA) cells showed no significant difference in any of our treatment groups, indicating no damaging effect on either the epithelium or the stromal cells at the tested treatment intervals.

In summary, the evaluation of the immunohistochemistry of the canine eyes showed no negative effects of CAP treatment on corneal epithelial and stromal cells. The reaction to CAP seems to vary notably between tissues (45, 48). Weiss et al. (45) showed a dose-dependent effect of CAP on human fibroblasts of the foreskin. The dose was defined by the application time (3, 10, 30, 60, 90, 120 s), the distance was 7 mm (45). A reduction in cell proliferation of more than 70% was observed for all CAP exposure times compared to controls (45). CAP treatment times between 10 and 120 s appeared to inhibit cell growth with consistent effect, except for a treatment time of 3 s, which showed an increased cell number after cultivation again (45). With CAP treatment of 60 s, the number of cells in G1 of the cell cycle was highest compared to the control (45). This was interpreted as a cell cycle arrest or DNA repair phase (45). After each treatment time, visible changes occurred in the cytoskeleton of the cells. To sum up, the dosage correlated with generation of radical species with cell viability and antiproliferative effects (45). This disparity in the results can be explained by the different methods. In our study, the effects of CAP were investigated in a three-dimensional tissue model, whereas other authors mostly used cell cultures (45, 48). This may in part explain different tissue reactions.

Current studies of oncology are focussing on the use of CAP as an anti-cancer therapy (48, 49). Kugler et al. (49) showed significant pathologies after 60 s CAP application to neoplastic incubated chicken embryos. They found, that CAP application increased the intratumoral apoptosis rate, caused thrombosis of tumor vascularisation and reduced intratumoral vascular density (49). These cell-toxic damages of CAP cannot be replicated in ocular tissue with our study. One reason for this could be the different settings, like the choice of plasma source, the distance between the plasma and the tissue target and the dosage of plasma. The penetration depth of CAP appears to be the most important factor for cellular changes (48, 50). In human mucosal cell culture the penetration depth of CAP was defined with 270 μm (50). If these data are transferred to the cornea, the basal area of the stroma is not reached efficiently with CAP. Nevertheless, penetration of the epithelium and indirect cellular effect of the ROS and RNS may also reach deeper tissue. The stroma was also treated directly with CAP in the keratectomy area. In our evaluation, there are no differences between the stroma below the keratectomy and the stroma that was epithelialized continuously.

All in all, we found that all corneal defects were healed after 72 h of cultivation. A 5-min application of CAP resulted in a lower perilimbal epithelial cell density and fewer Ki-67 positive stromal cells compared to a 2-min treatment, although the significance of these findings is unclear. There were no differences observed in apoptosis or fibroblast activity.

Limitations of the study are the small number of canine corneas (*n* = 20) available during the study period and the short cultivation time of 72 h. This allowed the analysis of short-term effects only. The acquired data is only applicable to the kINPen^®^ VET plasma pen and other plasma sources may have other effects on the corneal tissue.

It is unclear whether results of the *ex-vivo* culture can be extrapolated to the *in vivo* situation. However, our results support a clinical study in patients with corneal ulcerations.

A prospective clinical study is necessary to assess safety and efficacy of CAP application on the ocular surface in dogs. Different CAP treatment protocols with regards to dosage, duration, and distance of application should be explored in more depth. The evaluation of long-term effects in an extended cultivation phase will also have to be investigated. Further *in vitro* testing is needed to especially assess the impact of CAP on limbal stem cells in more depth, given their dominant role in corneal epithelial wound healing.

In conclusion, CAP treatment using the kINPen^®^ VET plasma pen for durations of 2 or 5 min does not interfere with the healing of canine corneal epithelial wounds in an *ex vivo* ALI culture. Given its notable antimicrobial properties, CAP presents a promising avenue for future therapeutic strategies in the management of infectious keratitis.

## Data Availability

The raw data supporting the conclusions of this article will be made available by the authors, without undue reservation.
